# Dr. Sutton on the Cure of Rheumatism

**Published:** 1813-11

**Authors:** Thomas Sutton

**Affiliations:** Greenwich


*To the Editor of the Medical and Physical Journal.*

SIR,

IT may appear somewhat to border on presumption, to undertake to maintain opinions contrary to the tenour of one of the most respectable publications on a medical subject that I am acquainted with, whether we view it in regard to style, or the candor with which it is published, or the well-deserved reputation of the author, supported also as it is by some present authorities of deserved influence in the medical world: I refer to Dr. Haygarth's second edition on the subject of Acute Rheumatism. Such a weight of authority that has now engaged in this cause, and adopted the practice which is the particular subject of the volume, must tend much to fix those who may have been wavering in their opinions respecting the cure of that disease, and also those who can have little else to guide them, beyond a fair application of the practice which they are taught through lectures, or which they learn from books. For myself, I should have been very happy if I could have received information through so respectable a channel as the present author, which had perfectly convinced me. But as that is not the case, I feel more inclined to hazard my feeble opinion in a somewhat contrary direction to this respectable publication.

We have very plainly seen, in the instance of Sydenham, in what strong chains he has held the medical world, in respect to his opinions on the Gout, which was one of the last of his performances, when his reputation had arrived at the highest pitch of credit. And in the same way we may be likely again to be bound down for many years to a practice coming in the shape of most overwhelming authority.

The reputation of Dr. Haygarth has deservedly arrived at the highest degree of eminence; and if with such reputation he promulgates opinions, they are likely to make the deepest impression. My experience has, however, led me to conclusions different from the publication alluded to; and and these conclusions, as they appear to me important, under the present state of the question, I shall most freely impart.

I have for many years, at various times, adopted the practice which Dr. Haygarth recommends; but I am sorry to say I could never conclude very favorably of it. It must not, however, here be understood, that I have seen much evil ensue from it; but it has never clearly appeared to me to be followed by any decidedly salutary effects. I am persuaded it may happen, that cases of acute rheumatism will be very short under various modes of treatment; but this does not prove the exact efficacy of any of these plans as a remedy for the disease generally.

Dr. Haygarth has given a case of a quick recovery from this disease, by the use of the Peruvian bark; and this very patient had been at some previous time ill of the same disease, under a different treatment, for many months. I will give a parallel case, under a different method of cure.

A robust seafaring man had been taken with a shivering, followed by an attack of fever, with pains in his limbs, three days before I saw him When I visited him, he was in the most helpless state imaginable, with rheumatism, and swellings in every limb and almost every joint. Two years before this attack, he had suffered from the disease through fifteen months, which incapacitated him for any employ during that time. He had not been bled in the first attack; but I now directed twenty ounces of blood to be immediately drawn; he was also smartly purged with neutral salts. I saw him two days after this, he was much better. I however again directed blood to be drawn, and that he should again be purged. I visited him two days after this, when I found him up, with very little rheumatic pain; and in the course of a week he came to my house, a walk of some distance, and informed me he intended immediately to go to work.

This was as severe a case of acute rheumatism as I ever saw, and was as rapidly cured. If, indeed, we could always succeed so well in the cure of this disease, we might be said to have found out a certain remedy for it; but since this, I have never seen so quick a recovery from the same means, and therefore I do not think the plan pursued in this case entitled to be considered as a guide for the cure of this disorder generally. I think, also, I have seen very beneficial effects to follow perspirations; but they by no means frequently carry off the disease, and therefore I should judge sudorifics not to be always actual remedies for acute rheumatism.

I am very glad Dr. Haygarth has given this edition of his

 book book to the public, because I think he has been more explicit in one important point than he was in his former edition, at least so I am impressed, for I have not his former work by me. The tenor of that, in so far as my mind is impressed, was rather to dissuade from blood-letting, and to trust to the use of the bark. At present I am pleased to find that the bark is to be left off, in some cases, to give way to blood-letting, and after a time to begin it again. In regard to blood-letting, in so far as my opinion goes, it appears to me to be almost indispensable: I do not say that it is a positive remedy for the acute rheumatism, but I think it ought almost always to be used to prevent some of the serious consequences of the disease, if we were not quite certain that it would contribute towards its cure.

Early in my medical career, I happened to be placed in a neighbourhood where the prevailing practice in this disorder was that of copious bleeding and purging, with antimonial and saline medicines. I do not say that by this mode of treatment the diseases were not often very obstinate, but they certainly maintained the character of being less inimical to life than I have found them of late years; and, indeed, according to Dr. Haygarth's statement, acute rheumatism is full as fatal as fever, if in addition to this we take into the account those who are attacked afterwards with diseases of the heart, which are very numerous. I had the misfortune to be called to three rheumatic patients within a very short space of time, who died of the rheumatic phrenitis, and all of whom might with propriety have been bled. I am strongly impressed that these events would not all have happened, had that operation been gone through. I have also seen numerous cases of rheumatism of the heart, and only in one instance could I discover that the parties had been bled, and that one in no considerable degree. My experience, therefore, leads me to infer strongly, that these fatal events and serious diseases would have been mostly avoided by the liberal use of the lancet, or other means of losing blood. I am, therefore, as I say, glad to find Dr. Haygarth keep in view and recommend the remedy of blood-letting, more than his other publication appeared to me to do.

One evil arising from the use of the bark, in London and in its vicinity, is, I fear, the almost total neglect of the lancet in this disorder, which I trust will be more revived. Theoretically, in fact, there seems to be a contradiction between the remedies of the bark and blood-letting, and I am persuaded this seeming opposition has had great eff t in preventing the use of the lancet.

Having

Having said as much as may be necessary at present on the subject of blood-letting, in so far as Dr. Haygarth and myself are concerned, I must now advert to antimonials and sudorifics. I consider, in an early stage of the complaint, that it is desirable to procure perspiration, nor is that difficult. With this intent antimonials, joined to saline medicines, may be very proper; for, as I have said before, I believe I have seen cases in which perspiration occasioned very material relief, though these are not numerous: but I have never seen perspirations continued for a long time attended with any advantage, if obvious benefit has not ensued within the first twelve hours; but when they have been protracted, they have appeared to be decidedly detrimental, and therefore not to be encouraged. The use of antimonials, therefore, beyond this period, seems to me to be of no other advantage, than in so far as they render the bowels soluble; but in so far as they encourage perspiration, by no means desirable: in fact, it has been my practice for some time absolutely to discourage them, by recommending any flannel wrappings to be laid aside, by prohibiting any warm drinks, and by desisting from the further use of antimonials. To curb this state of perspiration, I confess I have not seen the bark sufficiently effectual, and I am certain I have often seen it increase the inflammatory symptoms, and materially aggravate the pain. I might perhaps allow, that in such circumstances small doses of bark may be less detrimental than sudorifics; but that is all the praise I can give that medicine.

In regard to the use of anodynes, I can say nothing, from my own experience, that warrants me to conclude favorably of them generally, though I have occasionally seen some good effects; but from what I have observed, if 20 drops of laudanum is not attended with evident advantage, no better success will generally ensue from a larger dose, but frequently an exacerbation of the night paroxysm to a considerable degree. I have for many years agreed with Dr. Haygarth, and Dr. Currie of Liverpool, that acute rheumatism is one of the most tedious and intractable of diseases: I feel inclined, however, of late, much to modify that opinion, and to a certain degree to reject it.

The means of cure of acute rheumatism which I have hitherto mentioned, have appeared insufficient to satisfy me that something further ought not to be attempted. In respect to the management of the patient, in discouraging perspirations I considered that I had gained something, and therefore I not only recommended every local means to be avoided that were likely to increase that secretion, after a time time alluded to, but also to abstain from such medicines as might promote it. Antimonials and saline medicines have been therefore, recommended to be laid aside; and the vitriolic acid, in the form of Infusum Rosæ, to be given. Epsom salts have occasionally been added to this; or if the bowels happened to be open enough, or other aperient medicines employed, six or eight grains of nitre were directed to be given with each dose. In this way I was better satisfied to conduct the treatment of the disease than by the use of antimonials or the bark. Lastly, I advanced another step, which was to adopt the use of local cold, which has been effected by camphor mixture, and which I was principally induced to use on account of the advantages derived from local cold in peritonitis, as stated in my tract on that disease.

This then, in addition to the other means which my observation has taught me to approve, appears to conduct the disease to a speedy and safe conclusion; and they altogether seem to me to be well entitled to be considered as positive remedies for acute rheumatism.

Under the supposition, therefore, that I was called immediately on an attack of this disease, I should recommend blood to be drawn to some extent, to open the bowels, and to give antimonial and saline medicines. On the following day, if the symptoms of general fever were not abated, I should again direct the use of the lancet. If the antimonial and saline medicines had produced copious perspiration without material relief, I should, on the third day, order them to be desisted from. I should now recommend the vitriolic acid, with constant attention to the bowels, and the use of camphor mixture to those joints which were the most painful; and all along prohibit the use of flannel and hot applications to the pained parts, or the warm bath; and to keep the body only moderately covered. I have been better satisfied with the use of a dose of four or five grains of Pil. ex Aloe cū myrrh every six hours, and an occasional purgative of a more active quality, such as castor oil or salts, by which the bowels are kept in a sort of constant activity, after the first days of the attack, than by the use of occasional active purgatives. Through this mode of proceeding I find very little difficulty in subduing acute rheumatism in its ordinary form, and I am able to state confidently a material change for the better in a week or ten days, if not a total subjugation of the disease.

I shall now mention some of the last cases of this disorder that have been conducted upon this plan.

*Case 1st.*—A person of about 35 years of age had been eight days ill of acute rheumatism. He had been bled in the the commencement of the attack, the bowels opened, and saline antimonial medicines given. He had also taken some opium without relief, and was much wrapped round with flannel. During this time his pains had not abated, but the perspirations had been profuse, and he had been in a state which his friends thought approached to delirium on the night preceding my visit, and was not quite collected when I saw him. As I have a great dread of rheumatic delirium, I did not feel easy for the event of this case. I, however, directed all the flannel wrappings to be laid aside, sixteen ounces of blood to be taken from the arm, and a medicine composed of Infus. Rosæ, with a sufficient quantity of Magnesiæ Sulphas. On the next day the patient was somewhat better; had passed upon the whole a better night. I, however, judged he ought to lose more blood to the extent of eight or ten ounces, the other medicine to be persevered in; and, as the patient mentioned something of a cold application, I directed camphor mixture to be employed to the parts most in pain. On the following day my patient was much better, had passed a good night, and was in very fair progress in amendment. In four days after this, being a week from my first visit, I found him dressed, and rapidly recovering, which was afterwards uninterrupted.

*Case 2d.*—A woman who had suffered from an attack of that species of puerperal fever which I gave an account of in the Edinburgh Medical and Surgical Journal, and who had recovered under Mr. Butler's care, at Woolwich, in the manner I had described, was, about two months after that attack, seized with acute rheumatism. When I saw her she was in the most excruciating pain. She was much wrapped round with flannels, and in the most profuse sweat. The disease occupied the whole of her limbs completely. As she had suffered from diarrhœa under the puerperal attack to a considerable degree, it was not thought right to bleed her. The whole of the flannel wrapping was directed to be laid aside, and the camphor mixture to be applied to some of the pained parts first, and the bowels to be kept open by saline purgatives. I found from Mr. Butler, three days afterwards, that all my directions had been complied with, and that the patient was so much better as to be up and dressed, and in the course of a week she was so well as to entirely leave off medicine.

*Case 3d.*—A young gentleman, æt. 13, after being much heated by violent exercise, was taken, on the following day, with a cold shivering, succeeded by fever, with pains in his joints. On my second visit, I found the disease to be acute rheumatism, by the increase of pain, swelling in his knees

 and and ankles, and fever. As the swelling came on in the evening after I saw him, his friends wrapped them round with flannel, and by the aid of the saline mixture and James's powder he had been in a profuse sweat for sixteen hours, without any abatement of the symptoms. On my visit I directed the flannel to be discontinued, and the parts affected to be kept moistened with camphor mixture. Two large leeches were also applied on the back of each hand, which bled very considerably for many hours; and Epsom salts in Infus. Rosæ were directed to be taken. In two days the fever and pain were so abated that I found the patient up and dressed. A pill with three grains of Pil. ex Aloe cū Myrrh, to be taken twice a-day, was now directed; and the Infus. Rosæ with nitre. The patient had no further return of the disease, but continued to wrap the joints affected round with cloths, moistened with camphor mixture, for some time, at nights going to bed; and he was so sensible of the relief he experienced from this application, in comparison to his sensations under the flannel, that he was afterwards very desirous of using it. About a fortnight from this time I was desired again to visit the patient, who complained of some weakness in his ancles, and languor. Upon examining into his state of health, I found rather too much quickness of pulse, with a sharp stroke, and some palpitation at the heart. In the intervals of my visits, he had taken the bark in doses of ten grains three times a-day. I directed five grains of Pilula Ferri cū myrrh to be given twice a-day with some infusion of gentian, and the bowels to be kept open with castor oil, or equal parts of rhubarb and jalap; and the patient soon became better, and went to school; but, upon his again complaining of languor, he was sent to the sea side to bathe, where he most completely recovered.

*Case 4th.*—A gentleman, about 40 years of age, of a delicate frame, after visiting some damp apartments, was attacked with pain in the right shoulder, accompanied by a severe shivering and fever. Some years ago he had labored under acute rheumatism, and in one of the attacks had been confined six weeks. Latterly he had suffered some pains in his joints, which he had been able to remove by the help of a flesh-brush, which he applied also on this occasion, but without relief. He then had recourse to an old prescription, with volatile tincture of guaiacum and thirty drops of laudanum, to be taken at night, which had occasioned no relief. On the fifth day of this attack he sent for me. He had passed a very bad night; the shoulder was in great pain; he had a coated tongue, with much febrile heat, and a pulse of 108. He had also perspired very freely without relief, and had involved involved the part affected in flannel, which only caused an aggravation of his pain. He had taken some castor oil on the day I saw him, that had acted on his bowels, notwithstanding all which the disease was gaining ground. I directed the flannel to be removed from the part, and six leeches to be applied, and encouraged to bleed freely, which, on the following morning, I found took place. The Pilula ex Aloe cū myrrh, in four-grain pills, was directed to be taken every six hours, with a medicine composed of infusion of senna and barley water. On the next day the patient was somewhat better, but had still considerable pain. I now directed the camphor mixture, which was sparingly used, but the patient was relieved. His fever was gone, and only a considerable degree of stiffness in the part, and numbness of the whole limb. I still persisted in advising a more free use of the lotion, and on my visit at the interval of a day, (the fifth from my first seeing him,) I found he had in some measure complied, and was then capable of using a pen, and elevating the limb from the side at a considerable angle. The patient's expression now was, that every time he used the lotion it loosened the joint; and he found himself so well as to require no further attendance from me.

These then are the histories of the last cases of acute rheumatism that have occurred to me; and the quick recovery of the patients justify me at least in the conclusion, that the disease could by no known means have been more speedily removed; and, so far as my observation can guide me, I conclude that neither the bark, nor antimonials, nor any other plan of cure that has hitherto been recommended, could have succeeded so well. This, therefore, stamps its value. Future observation, perhaps, may lead to a still more speedy and efficacious method of treatment, which I shall be happy to witness.

This hasty account, which, however, I hope is such as to be completely intelligible, I have been thus induced to give at the present moment, when the medical world are likely to receive information on the subject of rheumatism with increased interest, on account of Dr. Haygarth's publication. As I cannot accede to the practice he recommends, I think it my duty to communicate my dissent with every respect. I trust I have stated nothing that will fail in other hands; and if it should happen so, I am sure Dr. Haygarth will be the first to excuse me in this attempt to invalidate his practice in rheumatism. If this should completely succeed, he has such reputation in store as ought to satisfy any one, and which is built upon an immoveable foundation, and has stood the scrutiny of time.

It might have been expected that I should before have taken notice of the author who has much preceded me in the use and recommendation of cold applications to the parts affected in the disease now under discussion; but I considered the most natural mode of statement to be that which connected the progressive stages by which I arrived at the treatment I now confidently recommend. Dr. Kinglake's book and opinions are before the public, which certainly settles his priority of claim in this respect. Without pretending to any discovery, my whole object is to put the public in possession of the most material observations I have made, through a series of years, towards the best mode of subduing acute rheumatism.

*Greenwich,* THOMAS SUTTON, M.D.

*Sept.* 25, 1813.

